# The translational challenge in Chagas disease drug development

**DOI:** 10.1590/0074-02760200501

**Published:** 2022-05-23

**Authors:** Jadel M Kratz, Karolina R Gonçalves, Lavínia MD Romera, Carolina Borsoi Moraes, Paula Bittencourt-Cunha, Sergio Schenkman, Eric Chatelain, Sergio Sosa-Estani

**Affiliations:** 1 Drugs for Neglected Diseases initiative, Geneva, Switzerland; 2 Universidade de São Paulo, Instituto de Ciências Biomédicas, Departamento de Microbiologia, São Paulo, SP, Brasil; 3 Universidade Federal de São Paulo, Departamento de Ciências Farmacêuticas, Diadema, SP, Brasil; 4 Universidade Federal de São Paulo, Departamento de Microbiologia, Imunologia e Parasitologia, São Paulo, SP, Brasil; 5 Epidemiology and Public Health Research Centre, CIESP-CONICET, Buenos Aires, Argentina

**Keywords:** Trypanosoma cruzi, animal model, *in vitro* assay, pharmacokinetic pharmacodynamic (PK/PD) relationship, dormancy, persisters

## Abstract

Chagas disease is a neglected tropical disease caused by the protozoan parasite *Trypanosoma cruzi*. There is an urgent need for safe, effective, and accessible new treatments since the currently approved drugs have serious limitations. Drug development for Chagas disease has historically been hampered by the complexity of the disease, critical knowledge gaps, and lack of coordinated R&D efforts. This review covers some of the translational challenges associated with the progression of new chemical entities from preclinical to clinical phases of development, and discusses how recent technological advances might allow the research community to answer key questions relevant to the disease and to overcome hurdles in R&D for Chagas disease.

American trypanosomiasis, widely known as Chagas disease (CD), is caused by the protozoan parasite *Trypanosoma cruzi* (*T. cruzi*) and considered a neglected tropical disease (NTD) by the World Health Organization.^1^ It was first described more than 100 years ago, but still represents a global public health problem and remains an endemic disease in Latin America. With an estimated 6-7 million people infected worldwide, causing around 7,500 deaths annually and life-long morbidity and disability, CD has an important economic and social impacts.^1^ ^,^ ^2^

*T. cruzi*, the etiological agent of the disease, is mainly spread by blood-sucking triatomine bugs and congenital transmission. The parasite can also be transmitted through blood transfusions, organ transplantation, and laboratory accidents, or orally, with an increasing number of outbreaks associated with contaminated food or drink.^1^ ^,^ ^2^ ^,^ ^3^

The disease presents two clinically distinct phases: (i) after infection, an initial acute phase characterised by high parasitaemia, usually with only mild symptoms or asymptomatic (although fatality rates can range from 2% to 8%); (ii) after 4-8 weeks, an adaptative immune response reduces parasitaemia to low or undetectable levels and, if untreated, the patients enter the asymptomatic chronic phase, the so-called indeterminate phase that continues for the duration of a person’s life. Around 30-40% of patients will progress to a symptomatic chronic disease with cardiac and/or digestive involvement, usually 10-30 years after the initial infection. The main factors associated with progression to the symptomatic chronic phase are still uncertain, and accurate prediction of disease progression remains challenging.^1^ ^,^ ^2^ ^,^ ^3^ ^,^ ^4^

In the absence of a prophylactic vaccine, a mixture of vector control practices, timely diagnosis, and treatment of patients is critical for disease control programs and can dramatically reduce the heavy burden caused by CD.^4^ ^,^ ^5^ ^,^ ^6^ Regarding treatment options, the only two approved drugs are the nitroheterocyclic compounds benznidazole (BZN) and nifurtimox (NFX).^7^ Although possessing a very clear trypanocidal effect in humans, these drugs are contraindicated during pregnancy, and prone to inducing adverse effects that lead to treatment discontinuation in 15-20% of patients, while ultimately, treatment’s capacity to prevent further progression of cardiomyopathy once it has already developed is still uncertain.^7^ ^,^ ^8^

These drugs were developed over 50 years ago, and since then very few clinical trials with new chemical entities have been conducted. Most studies in humans have focused on the evaluation of new treatment regimens of approved drugs [e.g., different BZN treatment dose and/or duration in the Benznidazole New Doses Improved Treatment & Associations (BENDITA trial)].^9^ The few examples of clinical trials that included new chemical entities as monotherapy (e.g., fexinidazole and posaconazole) or in combination with BZN (e.g., E1224 a prodrug of ravuconazole, belonging to a compound class targeting the ergosterol synthesis pathway) were unfortunately not successful.^9^ ^,^ ^10^ ^,^ ^11^ ^,^ ^12^ ^,^ ^13^ As will be further discussed in this review, despite the clinical failure of these compounds, information gathered from these clinical trials is continuously flowing back into the R&D process and helping improve models and their predictive capacity. Moreover, new technologies are providing scientists with new knowledge to evaluate different classes of compounds and new chemical entities at the preclinical stage of development, and compare them with the current standard of care; this might prove very useful for improving the translational value of actual *in vitro* assays and *in vivo* disease models.

It is widely recognised that drug discovery and development is a complex endeavor in all therapeutic areas, and attrition rates are high despite technological advances and global efforts. In order to increase the chances of success, ideally new chemical entities would progress through the drug discovery pipeline following a very clear set of progression criteria (summarised in the target candidate profile or TCP) and be assessed using well standardised and validated assays and models for decision-making purposes.^14^ ^,^ ^15^ ^,^ ^16^ When specifically considering CD drug development, although Drugs for Neglected Diseases *initiative* (DND*i*) has established a CD target product profile (TPP) in partnership with multiple partners,^17^ and published disease-specific criteria for early-stage development,^18^ however, there are still important knowledge and technological gaps that hamper the development of new treatments for CD patients.

This review covers key aspects of parasite biology, *in vitro* assays, and animal models that impact translational potential within the development pipeline of antiparasitic drugs for CD, and in parallel discusses the challenges and opportunities that lie ahead in this field.

***In vitro* - parasite biology and assays**

A drug discovery project that aims to develop new chemical entities (NCEs) usually starts with the identification of active compounds (hits) via high- or medium-throughput screening of synthetic libraries (including libraries of approved drugs in the case of repurposing efforts) and/or natural products. Following primary screening, hits are resynthesised and submitted to confirmatory assays, which can use the same primary screening assay, or a distinct, orthogonal assay. Once hits are confirmed, the project usually then progresses to the multi-parametric optimisation of the initial hits into lead compounds (i.e., hit-to-lead and lead optimisation phases) that might be selected as preclinical and clinical candidates.

Primary screening assays are used for quickly tracking large compound libraries, separating active from non-active compounds with a minimum of false negatives and ideally with a high statistical confidence. Confirmatory screenings aim at selecting true hits, removing false positives and, usually, determining the potency, selectivity, and *in vitro* efficacy (in the case of phenotypic screening assays) of hit compounds. The strategy used for primary screening and discovery of chemical starting points can largely be divided into: (i) phenotypic-based screening, which uses whole cell-based assays that enable the quantification of a desired cellular phenotype as a consequence of compound cellular activity, and (ii) target-based screening, which uses biochemical or biophysical assays that usually enable the quantification of compound interaction with a single, purified and previously validated protein target, measured through target binding, inhibition or activation.^15^ ^,^ ^19^

Target-based screening assays can offer valuable information on the molecular mechanism of action and facilitate downstream compound optimisation based on compound-target interaction knowledge, while phenotypic assays allow for interrogation of compound activity in physiological conditions, against virtually all druggable targets, while concomitantly evaluating compound permeability and distribution across cellular membranes and compartments.^20^ ^,^ ^21^ ^,^ ^22^ ^,^ ^23^

In the case of CD drug discovery, as well as for most parasitic diseases, phenotypic-based screening has historically been favored over target-based screening due to the paucity of genetically and chemically well validated targets, and due to the lack of translation from cell-free assays to parasite growth inhibition *in vitro* and/or *in vivo* (e.g., cruzipain, trypanothione reductase / synthetase).^20^ ^,^ ^21^ Furthermore, the phenotypic strategy has been relatively successful in CD drug discovery, resulting in the development of clinically useful drugs without prior in-depth investigation of the molecular target (BZN and NFX) or the discovery of new promising candidates that had their molecular targets subsequently deconvoluted (e.g., proteasome inhibitors or cytochrome b inhibitors).^14^ ^,^ ^24^ ^,^ ^25^ But, as will be further discussed in this review, and regardless of the strategy used for screening and early development, a better understanding of the translational potential of NCEs requires a deeper understanding of CD pathophysiology and the resulting models.

The *T. cruzi* life cycle comprises different morphological stages that adapt to variable environments within the insect vector and mammalian and human hosts, using distinct biochemical pathways and molecular components (Fig. 1). Therefore, the choice of parasite stage to be used in a particular cellular assay or target validation effort is very important and likely impacts the translation from *in vitro* assays to animal models.

Some laboratories have relied on drug screening assays using epimastigotes, which are the proliferating forms found in the vector midgut, because they grow axenically in liquid cultures and are suitable for simple viability assays. However, the relevance of these results is questionable as epimastigotes are quite different from the parasite stages living in mammalian tissues, particularly the intracellular amastigotes that are responsible for tissue parasitism and arguably cause the symptoms associated with chronic CD.^26^ ^,^ ^27^ ^,^ ^28^

**Fig. 1:**
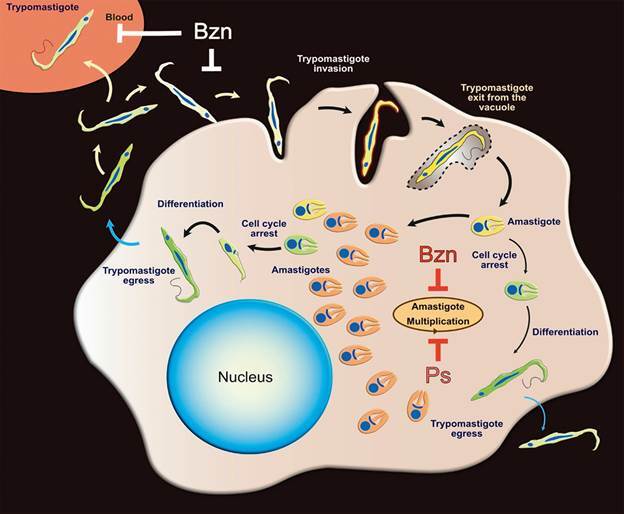
representation of *Trypanosoma cruzi* intracellular cycle in mammalian tissues. Trypomastigotes in blood interacts and invade mammalian cells forming a parasitophorous vacuole. The trypomastigotes exit the vacuole and transform into amastigote forms that start multiplying in the host cell cytosol (orange amastigotes). Following multiple rounds of division, amastigotes cease replication via cell cycle arrest (yellow to green amastigotes) and differentiate back into trypomastigotes that can egress and reinvade adjacent cells or circulate in the blood. The diagram also illustrates the existence of early cell cycle arrest in amastigotes (quiescent/dormant forms, light green) that can eventually differentiate back into trypomastigotes. Benznidazole (Bzn) and Posaconazole (Ps) inhibit primarily the intracellular multiplication of amastigotes.

Ideally, in a phenotypic-based CD drug discovery campaign (see Fig. 2 for a suggested screening cascade and progression criteria), compounds should be tested against intracellular *T. cruzi* amastigotes for initial hit identification and during the subsequent optimisation of chemical series, and the cytotoxicity against the host cells evaluated in parallel to ensure minimal selectivity. Additionally, important parameters describing absorption, distribution, metabolism, and excretion (ADME) properties, pharmacokinetics and safety can be evaluated concomitantly.

**Fig. 2:**
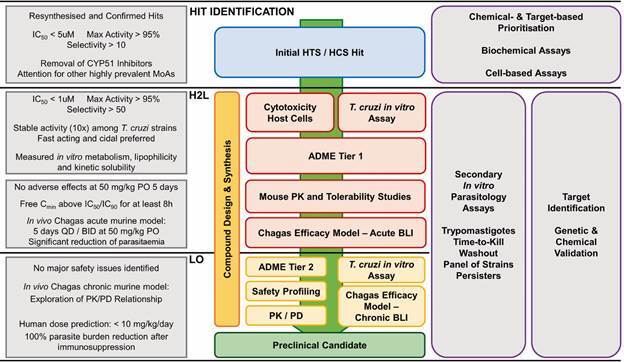
suggested screening cascade for the identification and progression of new chemical entities for Chagas disease. Assays used in different drug discovery stages (centre), with secondary profiling studies (right) and suggested progression criteria (left). HTS: high-throughput screening; HCS: high-content screening; IC_50_: half maximal inhibitory concentration; MoA: mechanism of action; PK: pharmacokinetics; ADME: absorption, distribution, metabolism, excretion; BLI: bioluminescent; QD: quaque die (once a day); BID: bis in die (twice a day); PO: oral dosing; PK/PD: pharmacokinetic/pharmacodynamic relationships; Free C_min_: free minimum plasma concentration; H2L: hit-to-lead phase; LO: lead optimisation phase.

Several groups around the world use a cell-based reporter assay that relies on colourimetric quantification of the product of beta-galactosidase activity expressed by a genetically modified *T. cruzi* clone of the Tulahuen strain.^29^ There are also reports of parasites expressing fluorescent proteins.^30^ Another variation of cell-based screenings used for anti-*T. cruzi* drug discovery is based on high content screening (HCS) assays. HCS is a sophisticated technology that enables quantification of parasite infection through automated image analysis of fluorescent microscopic images of parasites and host cells - usually achieved with simple DNA and whole-cell fluorescent stains or by deploying trypanosomes expressing fluorescent proteins (Fig. 3).^31^ ^,^ ^32^ ^,^ ^33^ ^,^ ^34^

**Fig. 3:**
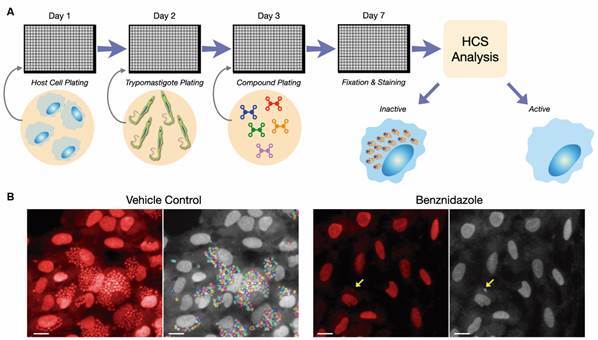
high content screening (HCS) for the discovery of anti-*Trypanosoma cruzi* compounds. (A) Schematic representation of a general HCS assay setup. Host cell lineage and *T. cruzi* strains of choice can vary significantly between laboratories and assays. Infected cells are exposed to compounds post-infection for a defined period of time and then antiparasitic activity is evaluated against intracellular amastigotes. Microplates are processed for image analysis. Highly active compounds will result in the (nearly complete) clearance of intracellular amastigotes. (B) Typical images of *T. cruzi*-infected cells treated with vehicle (left) and an efficacious concentration of benznidazole (right). Raw images are shown in red-stained host cell and parasite, and one key feature of HCS automated image analysis, amastigote segmentation and quantification, is shown in colored lines over grey-colored cells. While efficacious, benznidazole cannot often clear all intracellular amastigotes during short exposure times, and some amastigotes might remain after treatment (arrows).

While colourimetric assays have a fast, simple and relatively low-cost setup that can be accessed in low resource labs in developing countries, they have the disadvantage of being less sensitive than image-based screening, and can result in the selection of less efficacious compounds (unpublished observations). Conversely, HCS assays are more informative, providing data not only on antiparasitic activity but also on compound selectivity and cytotoxicity against host cells in a single assay.^33^ Image-based screening can also be performed as manual low-throughput assays, through visual inspection of Giemsa-stained or fluorescent slides of infected cells that are then manually scored for parasite and host cell quantification, with similar results, albeit with throughputs that are often not compatible with large compound library screening.

In modern drug discovery screening campaigns, confirmed hits and early-stage lead compounds are often submitted to a secondary parasitology assessment that combines target- and cellular-based assays (Fig. 2).^8^ ^,^ ^14^ ^,^ ^15^ ^,^ ^16^ This multifaceted approach allows the progression and prioritisation of compounds based on potency, mode of action, and molecular target. A typical step taken during secondary screening is the prioritisation of series that show broad spectrum activity against different *T. cruzi* strains. *T. cruzi* is a highly genetically heterogenous parasite, currently divided into seven phylogenetic groups (TcI - TcVI, and Tcbat). *T. cruzi* from all groups have been shown to infect humans, and different groups have been sampled at different frequencies across the Americas, but there is no clear correlation between geographical location and outcome of the disease. Also, *T. cruzi* groups are differentially distributed in regard to both human and sylvatic infections, depending on the geographical area (recently reviewed by Zingales).^35^ Furthermore, different *T. cruzi* strains have been shown to display varying degrees of susceptibility to BZN and NFX, both *in vitro* and *in vivo*.^36^ ^,^ ^37^ ^,^ ^38^ Often patients from endemic areas present mixed infection with *T. cruzi* from different groups.^39^ ^,^ ^40^ It is not known how, or to what degree, varying levels of drug susceptibility of different *T. cruzi* strains impact the therapeutic outcomes of BZN and NFX in CD patients. The limited evidence available would suggest that BZN presents variable levels of clinical efficacy in different geographical regions,^41^ which could be attributed to genetic differences between *T. cruzi* circulating in each area. However, the efficacy variability could also be linked to other confounding factors [e.g., age, pharmacokinetic (PK) variability]. It is practically impossible to confirm such hypotheses since seroconversion is the only available tool to assess clinical efficacy for CD (quantitative polymerase chain reaction - qPCR - is an important tool used in clinical trials but only provides an indication of treatment failure). Despite these uncertainties, it is suggested that the spectrum of anti-*T. cruzi* compound series in development are tested against different strains, ideally from distinct phylogenetic groups, in a standardised phenotypic screening assay.^42^ At the same time, it is important to highlight that information about compound performance against parasites with different replication rates [which can vary even between different clones of the same discret typing units (DTU)], and possibly against persisters forms, is becoming fundamental to understand the biological effects.^38^ ^,^ ^43^

Other properties evaluated during secondary parasitology profiling are the rate-of-kill and cidality of compounds. Series with faster rate-of-killing are associated with greater efficacy *in vivo* when compared to slow-killing compounds, as exemplified by BZN (fast-killing) and posaconazole (slow-killing); in fact, posaconazole (contrary to BZN) is not able to reduce intracellular infection to undetectable levels in most sensitive assays.^37^ ^,^ ^43^ ^,^ ^44^ This correlation between efficacy and rate-of-kill can be attributed to the mechanism of action: BZN is a drug that has a pleotropic effect, affecting different pathways, and is able to kill parasites regardless of their replicative state. BZN is active against both replicative (epimastigotes and intracellular amastigotes) and non-replicative (trypomastigotes) stages (at a higher concentration), while posaconazole is a drug that exerts its effect only on amastigotes that are undergoing division.^37^ ^,^ ^38^ Furthermore, rate-of-kill assays, such as the time-kill/time-to-kill assay, enable the evaluation of the exposure time and concentrations a compound requires to reduce *T. cruzi* infection to undetectable levels *in vitro*, a measure that can be useful in planning dosing regimens for *in vivo* efficacy studies.

These assays can also generate data on compound cidality, often in combination with washout/recovery assays, which measure the relapse of *T. cruzi* infection upon compound removal.^38^ There is limited published data on this kind of assay for series under development, but BZN, a cidal compound, can only achieve sterile cure *in vitro* in very specific experimental conditions, under very high drug concentrations (greater than 25-fold the EC_50_) and very long exposure periods (16 days of treatment). Also, prolonged posaconazole treatment in *in vitro* washout assays, even at high concentrations, does not provide total parasite clearance and relapse occurs relatively earlier than for BZN.^38^ ^,^ ^43^ However, how these *in vitro* regimens translate to *in vivo* conditions and further into treatment duration in the clinic is still a matter of intense debate.

Failure to eradicate *in vitro* infections has been attributed to the existence of persisters, which are parasites that can withstand high drug pressure for prolonged periods, and resume growth after drug withdrawal, a phenomenon that seems to exist in several microbes.^45^ ^,^ ^46^ *T. cruzi* persisters are thought to arise from non-dividing amastigotes, formed spontaneously both *in vitro* and *in vivo* (Fig. 1). Persistence is not due to development of drug resistance but rather tolerance to drug treatment as the new population of parasites growing after removal of drug pressure did not present a change in susceptibility to BZN *in vitro* in comparison with the parental population.^47^

The presence of quiescent/dormant forms has been described in other protozoan parasites. Those forms were linked with persistence in the host and drug treatment failure. Quiescent forms associated with a reversible growth-arrest phenotype have also been described in the hypnozoite of *Plasmodium* and the bradyzoite of *Toxoplasma gondii*.^48^ ^,^ ^49^

Other possible secondary profiling studies include the evaluation of compound series against (i) non-replicative trypomastigotes (either tissue-derived trypomastigotes and blood trypomastigotes), which have been used in an attempt to destroy circulating parasites and prevent cell reinfection; (ii) *T. cruzi* infecting different host cells; (iii) parasites harboring specific phenotypes and/or genotypes, such as resistance to a drug of interest.^38^ ^,^ ^50^ ^,^ ^51^ ^,^ ^52^ Although these assays provide useful information on the compound mechanism of action that undoubtedly enriches the understanding of their activity, further research is needed to establish their potential to predict the successful translation of series to both *in vivo* efficacy models and the clinic.

As previously mentioned, the molecular target(s) of compounds discovered through phenotypic-based screening is usually unknown and, although not strictly necessary for further development, knowledge of the compound target is highly desirable as it can facilitate optimisation of compound potency and selectivity, give information about potential safety issues, and may contribute to the development of drug combinations. For instance, chemical series that act on ergosterol biosynthesis through inhibition of *T. cruzi* sterol 14α-demethylase (TcCYP51) or inhibit the Q_i_ site of the mitochondrial cytochrome *b* (Cytb) are highly prevalent. These two proteins have been shown to be promiscuous targets, with an estimated 20-80% of confirmed hits emerging from *T. cruzi* primary phenotypic screenings reportedly targeting either TcCYP51 or Cytb.^50^ ^,^ ^53^ CD drug discovery portfolios with chemical series that target the same pathways are problematic, since they might fail altogether at a later stage, as has been the case with TcCYP51 inhibitors.^10^ ^,^ ^11^ ^,^ ^12^ Therefore, combining target-based and cell-based assays that can be used to de-prioritise particular targets is key and highlights the importance of back translation from the clinic to drug discovery.

To date, there are few examples of target deconvolution performed directly on *T. cruzi.* Target deconvolution is the process of translating the compound’s phenotypic activity into genetic and biochemical information, ultimately leading to the discovery of the molecular target(s). One notable example of target deconvolution is the identification of GNF6702 (a pan-kinetoplastid proteasome inhibitor), through a forward genetic strategy; it involved selection of *T. cruzi* epimastigotes resistant to early GNF6702 analogs, followed by whole-genome sequencing that identified point mutations in *TcPSMB4*, which encodes one of the proteasome beta subunits. The link between gene and phenotype was confirmed by demonstrating that epimastigotes ectopically expressing the mutated copy of PSMB4 were more resistant to GNF6702 than wild-type epimastigotes.^24^

In fact, a clear link between genetic and chemical target validation is not always easily achievable, especially in the case of *T. cruzi*.^54^ The first problem has been to better understand the genomic structure and variability of the different strains. The use of long pyrosequencing techniques has now produced a clearer picture of these variations.^55^ ^,^ ^56^

The second problem has been to demonstrate the essentiality of specific proteins and enzymes as gene inactivation is still challenging in *T. cruzi.* The parasite lacks RNAi machinery and replacement must be done in at least two alleles due to the diploidy of the parasite. In some cases, there are more than two copies of each gene and ploidy variations are frequently observed in the parasite population.^55^ ^,^ ^56^ Conditional knockouts using inducible expression systems represent an alternative approach, but so far robust regulatory systems are restricted to certain *T. cruzi* strains.^57^ Furthermore, genetic modifications are mainly developed for epimastigotes and for testing gene function in the amastigote stage; the parasites have to be transformed into infective trypomastigotes, a process that is often not achievable with several of the modified strains (our unpublished observations). In some cases, deletion of one allele decreases viability, which provides evidence of essentiality.^58^ More recently, CRISPR/Cas9 technology has been used to generate gene knockouts in *T. cruzi.* As this technique induces breaks in all alleles, it is common to observe failure in the generation of knockouts, which could indirectly indicate essentiality and, thus, suggests that the particular target is required and could be used in the development of drugs against *T. cruzi*.^59^

***In vivo* - animal models**

Animals models of disease play a key role in both basic and applied research. They not only help improve understanding of the pathology and etiology of a given disease, but also bring more confidence into the drug development process when moving NCEs forward into proof-of-concept Phase 2 clinical trials in humans. To be useful and have real translational value, animal models have to fulfill specific and well-defined criteria, not to mention be designed to answer specific questions (incorporating the relevant endpoints and biomarkers when available at that stage), have an adequate level of validation and reproducibility, and reproduce to some extent key characteristics of the disease in humans while conforming to current ethical and reporting guidelines.^60^

A thorough look at animal models of CD shows the striking plethora of species -from Zebrafish, to mice, rats, dogs to non-human primates (NHP), and models with a variety of endpoints that have been used by the research community as extensively reviewed.^61^ ^,^ ^62^ It also highlights the need for much better reporting of study data generated in these CD models^63^ and a clear need for fit-for purpose and harmonised animal models and experimental design protocols.^62^ An attempt at harmonising animal models for CD drug development was made a decade ago, following a meeting with experts in Chagas experimental research; it resulted in the publication of a proposed protocol for the testing of putative anti-*T. cruzi* drugs *in vivo* in animal models.^26^ Since then, there have been tremendous developments both in terms of better understanding and new knowledge of host-parasite interactions and infection dynamics as well as the rise and use of new imaging technologies and genetically modified parasites, making the “*Romanha protocol*” out of date. In 2008, Hyland and coworkers had already described the imaging of luminescent *T. cruzi* parasites in mice following infection and followed their dissemination to different sites during a 25-day infection.^64^ Transgenic parasites coupled with imaging technology provided a new tool for studying a number of aspects of CD, including rapid screening of potential therapeutic agents, roles of parasite and host factors in the outcome of infection, and analysis of differential tissue tropism in various parasite-host strain combinations. Later on, a very robust and informative murine model of *T. cruzi* infection using bioluminescence imaging (BLI) with a red-shifted luciferase transgenic *T. cruzi* parasite of the CL Brener strain (TcVI) was developed.^28^ Major new information came from these new developments and increased our understanding of parasite infection. *T. cruzi* infection can now be followed in real-time and the different stages reproduced, from the acute stage (characterised by very high levels of bioluminescence in all organs), to entry into the chronic stage where parasite burden is very low (characterised by much lower bioluminescence). Chronically infected mice developed myocarditis and cardiac fibrosis, despite the absence of locally persistent parasites in the heart. Interestingly, infection in the chronic stage showed a very dynamic spatiotemporal and focal distribution of parasites, not localised specifically to the heart and possibly other organs as had often been previously speculated. *T. cruzi* parasites were found to move rapidly from one site to another. The only sites where *T. cruzi* infection was consistently observed were reservoir sites in the gastro-intestinal tract, specifically the colon and stomach. In short, BLI has allowed a link to be established between parasite persistence and the pathogenesis of Chagas heart disease, and a better understanding of the association between persistence, pathogenesis, and immunity, which may help to optimise treatment.^65^ ^,^ ^66^ ^,^ ^67^

Moreover, the use of BLI allows investigators to follow the same mouse in time and reduce the total number of animals needed per study, in line with the 3Rs principles and ethical considerations when performing experiments with animals. These studies led to the development and validation of a new murine model that is very useful for assessing the efficacy of new compounds to provide parasitological cure (see Fig. 4 for a schematic representation of a chronic efficacy experiment).^68^ ^,^ ^69^

**Fig. 4:**
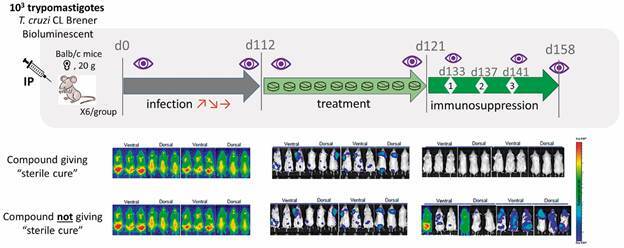
bioluminescent *in vivo* mouse Chagas disease model general scheme for compound efficacy assessment. N: imaging; dX: X days post-infection; t: round of immunosuppression X.

NHP are very often considered better models of disease and thus obligatory for moving compounds forward. However, results from NHP models for CD are controversial and, so far, there is no validated NHP model for CD. For that reason and considering costs, availability and ethics, Chagas murine models using BLI should become the new standard in the CD community and a standardised protocol as described in Chatelain & Scandale should be recommended.^62^ Whenever possible, associated blood sampling should be considered during in-life experiments to allow direct pharmacokinetic/pharmacodynamic (PK/PD) analysis in infected animals. In addition, considering the lack of biomarkers of treatment efficacy in the clinic, research on biomarkers of cure should be integrated earlier in the discovery process for potential translation to human in clinical trials. There is no doubt that further technological developments in the field such as double transgene *T. cruzi* and CRISPR/Cas9 will further increase the arsenal of methods to allow a better understanding of CD and host/pathogen interactions. Further increased confidence in CD *in vivo* animal models and their translational value will be achieved through back-translation of clinical data for new compounds that move into proof-of-concept phase 2 clinical trials and beyond (pending feasibility of treatment efficacy assessment).

**Challenges and future outlook**

There are still major challenges in the search for new drugs to treat CD. Very few clinical trials with NCEs have been conducted to date. It has therefore been largely impractical to evaluate the translational potential of models and assays included in the current screening cascades used in CD drug research and development.

Historically, most efforts in CD drug discovery have been focused on antiparasitic drugs, instead of pursuing an “antichagasic therapy”. This strategy is based on the assumption that removing all parasites from the body will halt disease development and/or progression, and its universality is likely associated with the fact that the tools available to this point mostly allow the assessment of antiparasitic activity (or proliferation of parasites) at least *in vitro* and *in vivo*. CD is a chronic and silent disease, with a complex pathophysiology; the scientific community is still struggling to understand key aspect of host-parasite interactions and has not yet been able to clarify why only a fraction of *T. cruzi* infected patients develop symptoms in the long term. Assays and biomarkers that enable prediction of disease progression are simply not available, and, therefore, a drug discovery campaign aiming at the identification of compounds that can avoid cardiac and/or digestive involvement in CD is not truly feasible today.

Even when considering the development of antiparasitic treatments one must accept that there are still knowledge and technological gaps. One critical issue is the lack of biomarkers of parasitological cure. Since seroconversion can take years in adults and a negative qPCR result is solely indicative of treatment failure, and therefore cannot be considered a surrogate of seroconversion, clinical assessment of efficacy is difficult. A test using validated surrogates of parasitological cure that would enable, in a timely manner, efficacy assessment of clinical candidates in patients (and ultimately supports regulatory registration) and in parallel helps assess the translational value of current models, would represent an exceptionally valuable resource for the Chagas community both at the R&D level and for patient counselling following treatment. The development of such a test is regarded as a key priority in the Chagas field and a TPP to guide its development has been recently published.^70^

The link between parasite persistence and cellular reinvasion, host immune response, and the pathogenesis of Chagas heart disease is now widely accepted.^28^ As a consequence, most anti-*T. cruzi* drug discovery projects use sterile cure (removal of all parasites from blood and tissues) as a criterion for progression. Some of the *in vitro* assays and animal models that allow such assessment are discussed in this review. However, ultimately, it is not yet clear what the main driver(s) of efficacy are, what compound properties are required to achieve such a high level of parasite clearance (especially considering parasite reservoirs and infection dynamics), and how the information obtained with these models will translate into clinical efficacy. Therefore, it is important to combine multiple endpoints and strategies that shed light on parasite localisation and persistence, drug potency and selectivity, mode of action, and the drug metabolism and pharmacokinetic (DMPK) profile of compounds. Only with multiple endpoints will the research community generate the data necessary to optimise candidates and understand their behavior *in vivo* (PK/PD relationships), refine models (including supporting biomarker validation), and possibly develop new treatments.

The recent description of dormant/quiescent/persister forms of *T. cruzi* and the potential association of this characteristic with treatment failures has highlighted once again that new knowledge can open up new opportunities.^45^ ^,^ ^46^ Although this parasite behavior could possibly represent an additional barrier for drug discovery, the development/validation of new assays might allow the exploration of other highly hypothetical treatment approaches, such as “parasite awakening”. Eliminating the parasite reservoir in immune privileged tissues might help to achieve sterile cure in humans via a combination of different modes of action, for instance.

In fact, CD drug discovery may benefit from the extensive use of front-line technology, such as the CRISPR/Cas9 system, proteolysis targeting chimeras (PROTACs), artificial intelligence and machine learning, and DNA-encoded chemical libraries. The systematic application of these new technologies might open new exploration venues that complement currently used methods and practices.

In summary, scientists will eventually make breakthroughs and new knowledge will arise. Hopefully, this knowledge will be readily applied to assay/model development and enable the progression of new candidates into clinical development, possibly supported by new technologies. Regardless of whether these campaigns are successful or not, it will be extremely important to back-translate this information into drug discovery and regularly revisit the screening cascade and TCP/TPP to reflect the latest developments. Only with such reiterative cycles of test-learn will it be possible to overcome the translational challenge in CD drug development.

As previously mentioned, most of the discussion in this review was focused on the development of NCEs as antiparasitic agents, but there are multiple groups pursuing other approaches such as the development of prophylactic / therapeutic vaccines, host-directed therapies, and treatments specifically designed for CD cardiomyopathy. In fact, other treatment modalities that have not yet been systematically explored in the field of CD, but are already consolidated in other therapeutic areas, might also represent a valuable opportunity, including, but not limited to, the use of drug combinations, monoclonal antibodies, and oligonucleotides.

**Concluding remarks**

Recent years have brought significant advances in the development of antiparasitics for kinetoplastid diseases. The approval of fexinidazole as the first all-oral treatment for sleeping sickness and the emergence in the leishmaniasis portfolio of at least six preclinical/clinical candidates in development are key examples of the progress made in translational research in the field of NTDs during the last decade.

The CD pipeline, however, remains much less populated, with just a few classes of compounds showing promising results and possibly advancing to clinical trials in the coming years. Back translation from clinical trials into drug discovery, together with the technical advances discussed in this review, will continue to contribute to a more favorable landscape. These might allow not only a better understanding of CD and an improvement in the translational value of the models currently used throughout the discovery pipeline but also lead to the development of new and more adequate assays in the future and an improvement of the current screening cascade and TCP.

Key questions still remain unanswered in CD. It is, therefore, of utmost importance for the R&D community to keep striving for a better understanding of the pathophysiology of the disease. Novel tools that allow progression of potential candidates with more confidence and establish their clinical potential in patients are also highly desirable. Assays that elucidate the role of dormant/quiescent parasites, for example, as well as the identification of biomarkers allowing the assessment of parasite clearance or prediction of disease progression will be welcome developments in the future.

Finally, it is important to highlight the value of multidisciplinary collaboration and broad sharing of information and tools. The CD drug development field continues to suffer from limited resources and often uncoordinated efforts within the Chagas community. The use of harmonised and well validated models, complementary approaches, and new technologies, together with a collaborative attitude in the space of this neglected disease, are essential components of a successful strategy to make new treatments available to CD patients.
